# Cellular Promyelocytic Leukemia Protein Is an Important Dengue Virus Restriction Factor

**DOI:** 10.1371/journal.pone.0125690

**Published:** 2015-05-11

**Authors:** Federico Giovannoni, Elsa B. Damonte, Cybele C. García

**Affiliations:** Laboratory of Antiviral Strategies, Biochemistry Department, School of Sciences, University of Buenos Aires, IQUIBICEN-Consejo Nacional de Investigaciones Científicas y Técnicas, 1428 Buenos Aires, Argentina; German Cancer Research Center, GERMANY

## Abstract

The intrinsic antiviral defense is based on cellular restriction factors that are constitutively expressed and, thus, active even before a pathogen enters the cell. The promyelocytic leukemia (PML) nuclear bodies (NBs) are discrete nuclear foci that contain several cellular proteins involved in intrinsic antiviral responses against a number of viruses. Accumulating reports have shown the importance of PML as a DNA virus restriction factor and how these pathogens evade this antiviral activity. However, very little information is available regarding the antiviral role of PML against RNA viruses. Dengue virus (DENV) is an RNA emerging mosquito-borne human pathogen affecting millions of individuals each year by causing severe and potentially fatal syndromes. Since no licensed antiviral drug against DENV infection is currently available, it is of great importance to understand the factors mediating intrinsic immunity that may lead to the development of new pharmacological agents that can boost their potency and thereby lead to treatments for this viral disease. In the present study, we investigated the *in vitro* antiviral role of PML in DENV-2 A549 infected cells.

## Introduction

The intrinsic antiviral resistance response, unlike cytokine-mediated response, involves the actions of pre-existing cellular proteins to repress viral replication [1, 2]. This group of proteins with potent antiviral properties known collectively as “restriction factors", are constitutively expressed in cells but can also be induced by type I interferon (IFN-I). Promyelocytic leukemia (PML) protein has been shown to contribute to intrinsic and innate defenses against a broad range of viruses [3]. PML has the ability to form nuclear bodies (NBs) that serve as a hub for the interaction and modification of over 90 cellular proteins. PML-NBs are sub-nuclear structures associated with the nuclear matrix, which have been implicated in numerous cellular processes including transcription, post-translational modifications, oncogenesis, innate immunity, and several antiviral responses [4]. The composition of PML-NBs is heterogeneous and includes constitutively expressed essential constituents such as PML protein, IFN-stimulated Sp100 nuclear antigen, death-domain associated protein-6, and really-interesting-new-gene (RING)-finger proteins [5]. PML-NBs range in size from 0.2 to 1.2 μm in diameter [6] and their number and distribution per nucleus vary considerably between 5 and 30 PML-NBs depending on the cell type, cell cycle, and cell condition [7].

PML-NBs have been shown to be an important intrinsic restriction factor and also contribute to innate defense against a broad range of viruses in the absence of IFN induction. However, IFN-I treatment increases the number and size of PML-NBs and enhances its antiviral activity [8, 9]. In turn, many viruses encode products that modify or eliminate PML-NBs in cultured cells. Since their discovery, PML-NBs have been investigated for their role in the virus-host cell interactions of DNA viruses that must replicate in the mammalian cell nucleus [10, 11], but more recently attention has been drawn to their antiviral role against RNA viruses. It has been demonstrated that fibroblasts derived from PML-/- mice are much more sensitive to some RNA viruses, such as the rhabdoviruses vesicular stomatitis and rabies virus, and the arenavirus lymphocytic choriomeningitis virus. These and other findings implicate PML in an intrinsic antiviral response of the cell that targets not only DNA but also RNA viruses [12–14].

Several PML isoforms generated by alternative splicing from a single gene are designated PMLI to PMLVIIb. They share the same N-terminal region, which encodes the TRIM motif (TRIpartite Motif), but differ in their C-terminal region. The variability of the C-terminal part is important for the recruitment of specific interacting partners and for the specific function of each PML isoform [15, 16]. The implication of PML in antiviral defense against DNA and RNA viruses from different families has been demonstrated in cells stably expressing individual PML isoform or in cells depleted for PML by RNA interference [17]. In particular, it has been observed that PML III and IV impair the replication of RNA viruses like the human immunodeficiency virus type 1 (HIV-1), influenza and rabies virus [14,18–19].

Dengue virus (DENV) is an emerging mosquito-borne human pathogen included in the family *Flaviviridae*. The four DENV serotypes (DENV1-4) affect millions of individuals each year by causing severe and potentially fatal syndromes [20]. The virion particle has a single-stranded RNA genome of positive polarity that codes for a polyprotein, which is co- and posttranslationally processed into three structural proteins (capsid (C), membrane (M), and envelope (E)) and seven nonstructural proteins (NS1, NS2A, NS2B, NS3, NS4A, NS4B, and NS5). Since no licensed antiviral drug against DENV infection is currently available and the most advanced DENV vaccine candidate did not meet expectations in a recent large clinical trial [21], it is of great importance to understand the factors mediating intrinsic immunity which may lead to the development of new pharmacological agents that can boost their potency and thereby lead to treatments for this viral disease.

In a previous study we have shown the contribution of PML to cellular antiviral defense against arenavirus multiplication [22]. Here we have extended those studies to explore the antiviral role of PML in the *in vitro* replication of DENV-2 in human A549 cells.

## Materials and Methods

### 1. Cells and viruses

A549 (human lung adenocarcinoma) and Vero (African green monkey kidney) cells were grown in Eagle's minimum essential medium (MEM) (GIBCO) supplemented with 10 and 5% fetal bovine serum, respectively and 50 μg/mL gentamycin. For maintenance medium (MM), the serum concentration was reduced to 1.5%. The C6/36 mosquito cell line from *Aedes albopictus*, adapted to grow at 33°C, was cultured in L-15 medium (Leibovitz) (GIBCO) supplemented with 0.3% tryptose phosphate broth, 0.02% glutamine, 1% MEM non-essential amino acids solution and 10% fetal bovine serum.

Virus stocks of DENV-2 (strain New Guinea C) were prepared in C6/36 cells and titrated by a standard plaque assay in Vero cells.

### 2. siRNA and plasmid transfections

PML silencing was accomplished by using commercial siRNAs (sc-36283; Santa Cruz Biotechnology). A549 cells grown on coverslips in 24-well plates were transfected with 100 nM siRNA using Lipofectamine 2000 (Invitrogen). The sequence of the RNA used as control was 5′-GACCACAATTCTCGATATACAUU-3′.

The plasmid encoding PML IV isoform (pcDNA-PML IV) was kindly provided by Dr. Martin Monte (FCEN-IQUIBICEN, Buenos Aires University, Argentina). Plasmid (1 μg/ml) was transfected into A549 cells grown in 24-well plates by using Lipofectamine 2000 (Invitrogen).

Cells were infected at 24 h post-transfection at a MOI of 1. At 24 h post infection (p.i.), extracellular medium was harvested to quantify virus production and cells were processed for immunofluorescence or real time RT-PCR assays.

### 3. Real time RT-PCR

Total RNA was extracted by using TRIZOL (Invitrogen Life Technologies) according to the manufacturer’s instructions. Then, cDNA was generated by using murine reverse transcriptase M-MLV (100 U/μl, Invitrogen) and random primers. This cDNA was amplified by real time PCR using SYBRGreen (Roche) detection. The mix reaction volume was 25 μl including 2 μl of cDNA, DNA polymerase GoTaq (5 U/μl, Promega) and specific primers to amplify selected genes (Table 1).

**Table 1:** Primer sequences used for real-time RT-PCR assays.

Primer	Orientation	Sequence
TLR3	Sense	5′-AGTGCCCCCTTTGAACTCTT-3′
	Antisense	5′-ATGTTCCCAGACCCAATCCT-3′
RIG-I	Sense	5′-TGTTCTCAGATCCCTTGGATG-3′
	Antisense	5′-CACTGCTCACCAGATTGCAT-3′
TRAF6	Sense	5′-TGCCATGAAAAGATGCAGAG-3′
	Antisense	5′-AAGGCGACCCTCTAACTGGT-3′
IL-6	Sense	5′-TGTGAAAGCAGCAAAGAGGCACTG-3′
	Antisense	5′-ACAGCTCTGGCTTGTTCCTCACTA-3′
IFN-β	Sense	5′-TAGCACTGGCTGGAATGAGA-3′
	Antisense	5′-TCCTTGGCCTTCAGGTAATG-3′
PML	Sense	5′-TTGAGTAAACYRTGCTGCCTGTAGCTC-3′
	Antisense	5’-GGGTCTCCTCTAACCTCTAGTCCT-3′

Real time PCR was carried out with an initial incubation at 95°C during 2 min, followed by 35 cycles of 30 s at 95°C, 1 min at 58°C, and 1 min at 72°C and a final step of 10 min at 72°C. Amplification plots were analyzed with Opticon Monitor 3.1 software and the comparative threshold cycle (Ct) method was used to determine gene expression relative to the cellular gene *β-actin*.

### 4. Indirect immunofluorescence assay

Cell monolayers were washed with cold PBS and fixed in methanol for 15 min at -20°C. For PML detection, indirect staining was carried out by using monoclonal (PG-M3; sc-966) or anti-PML polyclonal (H-238) antibodies (Santa Cruz Biotechnology), followed by TRITC-labeled goat anti-mouse IgG1 or anti- rabbit IgG1, respectively (Sigma-Aldrich). Viral glycoprotein E was stained using anti-E monoclonal antibody (Abcam, ab9202) and FITC-labeled goat anti-mouse IgG2a (Abcam, ab97244). After a final washing with PBS, cells were stained with DAPI and mounted in a glycerol solution containing 1,4-diazabicyclo[2, 2, 2]octane (DABCO). Slides were examined using an Olympus BX51 microscope (standard fluorescence microscopy) or an Olympus Fluoview FV300 (confocal microscopy).

### 5. PML-NBs measurements

To quantify the number of PML-NBs per cell nucleus, their number was counted in digital images obtained by confocal microscopy. To determine the size of PML-NBs, a scale bar was imprinted onto images by using the Olympus Fluoview Software. This bar was used to set a pixel/μm scale in ImageJ software and the line tool was used to measure the longest dimension of PML-NBs.

### 6. Cell cultures treatments

Preparation of conditioned medium derived from A549 infected cells: confluent A549 cells were infected with DENV-2 as described above. Following incubation at 37°C for 24 h, the medium collected from these cells was ultracentrifuged at 46,000 rpm for 2 h at 4°C in a SW55 Ti Beckmann rotor. The supernatant was collected and applied to fresh A549 control cells during 24 h to examine the effect of soluble factors.

IFN-I treatment: A549 and Vero cells were treated with 2500 UI/ml of IFN-I (Sidus, Argentina) during 24 h and cells were fixed and immunostained as described above.

Janus-activated kinase (JAK) type I inhibitor treatment: A549 cells were treated with 10 μM of JAK Inhibitor I (CAS 457081-03-7, Santa Cruz Biotechnology) during 2 h before infection. Then, this treatment was extended after infection for 24 h more, when cells were processed for real time RT-PCR.

### 7. Statistical analyses

Statistical analyses were performed using GraphPad Prism software. Comparison of means was tested by one-way analysis of variance (ANOVA) with Dunnett’s posttest. Statistical significance was defined as p < 0.05.

## Results

### Effect of PML expression on infectious DENV-2 particle production

Several *in vitro* studies with DNA viruses have shown that depletion of PML protein enhances viral replication [23–25], while the exogenous expression of PML protein, in particular the PML isoform IV, is implicated in innate immunity by enhancing IFN-β production during a viral infection and causing significant reduction of viral particles production [26–28].

To analyze the role of PML in DENV-2 replication, we transfected A549 cells with siRNAs that targeted all PML isoforms (PML-siRNAs) or with small non-interfering RNAs (X-siRNAs) used as control. In an additional experiment, A549 cell cultures were transfected with a PML isoform IV encoding plasmid (pcDNA-PMLIV), and non-transfected cells were included as control.

**Fig 1:**
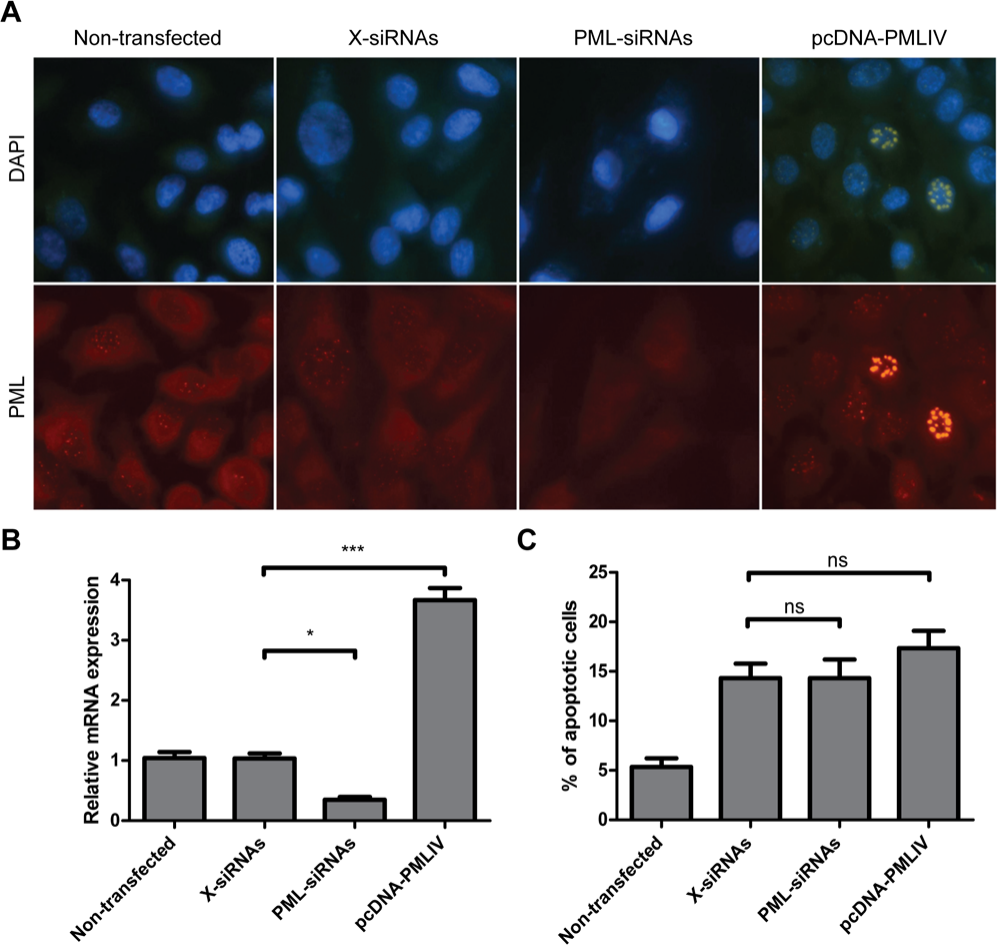
Assessment of PML silencing and overexpression in A549 cells. A549 cells were non-transfected, transfected with X-siRNAs, PML-siRNAs or pcDNA-PMLIV. (A) At 24 h post transfection, cells were fixed and PML protein was stained using anti-PML monoclonal antibody and TRITC-labeled anti-mouse IgG. Cells were visualized by fluorescence microscopy. Magnification: 400 X. (B) In parallel, cells were harvested for determination of PML-mRNA expression levels by real time PCR. PML-mRNA expression level is represented as fold difference relative to X-siRNAs-transfected cells and normalized to β-actin-mRNA. The reported values are mean ± SD (n = 3). Asterisks indicate a significant difference (*** p < 0.001; ** p < 0.01; * p < 0.05). (C) Counts of apoptotic cells and apoptotic bodies were performed using 400 X magnification. All identified apoptosis in the sample were counted and the % of apoptotic cells was defined as the total number of apoptotic cells and apoptotic bodies in at least 500 cells.

In order to assess the PML silencing by PML-siRNAs or its overexpression by pcDNA-PMLIV, the expression of PML protein was evaluated by immunofluorescence and the abundance of PML-mRNA was quantified by real time PCR (Fig 1A and 1B).

As shown in Fig 1A, the PML staining appeared as the typical discrete dots localized to nuclei (PML-NBs) and the level of PML-mRNA found in X-siRNAs transfected A549 cells was comparable to non-transfected cells (100 ± 14%) (Fig 1B). In contrast, in PML-siRNAs transfected A549 cells no evidence of PML expression was observed by fluorescence microscopy and only 35 ± 8% of PML-mRNA abundance was quantified by real time PCR.

On the other hand, the PML IV isoform overexpression was also confirmed by immunofluorescence and real time PCR assays. In these pcDNA-PMLIV-transfected cells, the immunofluorescence pictures showed cells with distinctively bigger size PML-NBs (Fig 1A) and the PML-mRNA level was considerably higher as shown by a 3.6 ± 0.4 fold increment of PML-mRNA in comparison to X-siRNAs transfected cell cultures (Fig 1B).

As it is well established that PML promotes apoptosis [29], and this event might have influenced virus yields, all the cell cultures were stained with DAPI and the number of apoptotic cells were quantified. Apoptotic cells were characterized by blebbing, cell shrinkage, with condensed hyperchromatic and beaded nuclear chromatin. It was observed a significant difference among the number of apoptotic cells between non-transfected cell samples and cells that were treated with lipofectamine, as expected. However, no difference in the number of apoptotic cells was observed among the samples that were transfected with X-siRNAs, PML-siRNAs or pcDNA-PMLIV (Fig 1C). These results suggest that silencing or overexpression of PML is not causing a secondary effect, reducing or increasing the apoptosis in these cell cultures.

Then, all cell cultures were infected with DENV-2 (MOI of 1) at 24 h post-transfection and were further incubated for 24 h when extracellular virus production was determined by standard plaque assay and viral antigen expression was analyzed by immunofluorescence. Also, a non-infected A549 cell culture was included as cellular control.

**Fig 2:**
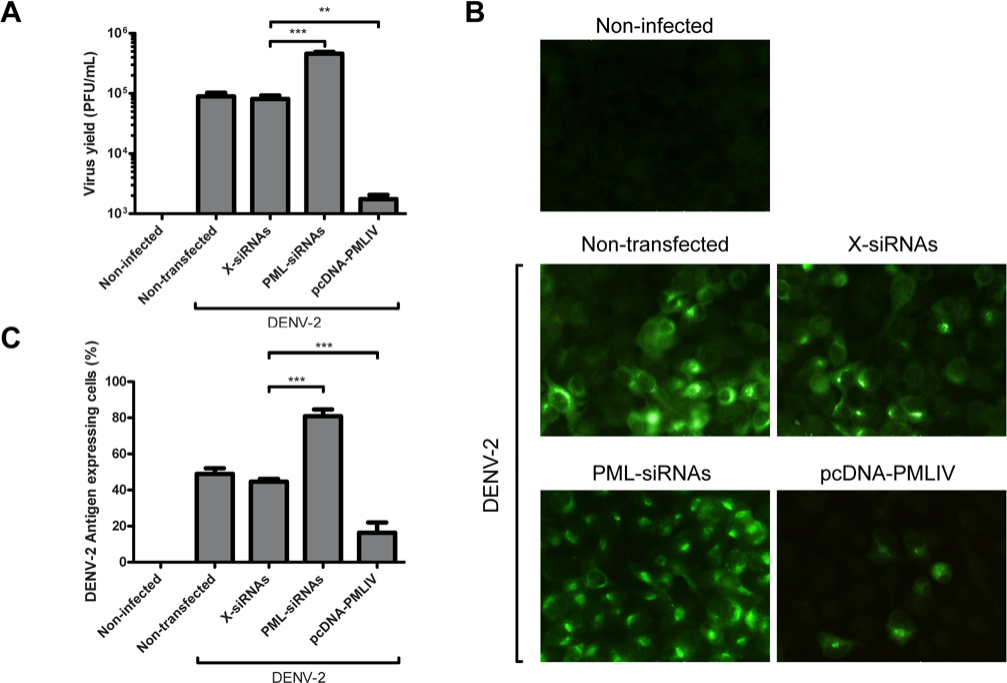
Effect of PML silencing and overexpression on infectious particle production and antigen expression. A549 cells were non-transfected, transfected with X-siRNAs, PML-siRNAs or pcDNA-PMLIV and infected with DENV-2. Non-infected cells were also included as a control (A) At 24 h p.i. viral yields were determined by a standard plaque assay. The reported values are mean ± SD (n = 3). Asterisks indicate a significant difference (*** p < 0.001; ** p < 0.01; * p < 0.05). (B) In parallel, cells were fixed at 24 h p.i. and viral glycoprotein E was stained using anti-E monoclonal antibody and FITC-labeled anti-mouse IgG2a. Cells were visualized by fluorescence microscopy. Magnification: 400 X. (C) Quantification of DENV-2 antigen expressing cells shown in B.

As shown in Fig 2A, the infectious particle production obtained from X-siRNAs transfected A549 cell cultures was similar to that obtained in non-transfected A549 cell cultures, with no significant difference in virus yields (8 10^4^ ± 3 10^4^ and 9 10^4^ ± 3 10^4^ PFU/ml, respectively). Interestingly, the silencing of all PML isoforms using specific PML-siRNAs caused 0.76 log increment in virus titre in comparison with virus yield obtained in X-siRNAs-transfected cells (from 8 10^4^ ± 3 10^4^ to 4.6 10^5^ ± 0,8 10^5^ PFU/ml). On the other hand, the PML IV isoform overexpression reduced significatively the virus titre, as 1.6 log (97.8%) decrement of DENV-2 extracellular production (1.8 10^3^ ± 0.8 10^3^ PFU/ml) was measured by standard plaque assay. These results were in accordance with the viral antigen expression observed by immunofluorescence at 24 h p.i (Fig 2B). The PML silencing rendered A549 cell cultures more susceptible to virus propagation as 81 ± 6% positive viral antigen expressing cells were observed in comparison with 45 ± 3% positive DENV-2 infected cells quantified in X-siRNAs-transfected control cultures. Noticeably, the overexpression of PML IV highly inhibited the viral antigen expression in these cell cultures (Fig 2C). It is worth mentioning that the antiviral effect was not restricted to DENV-2 as PML IV had antiviral activity against other DENV serotypes (data not shown).

### DENV-2 infection alters the intracellular localization and expression level of PML

**Fig 3:**
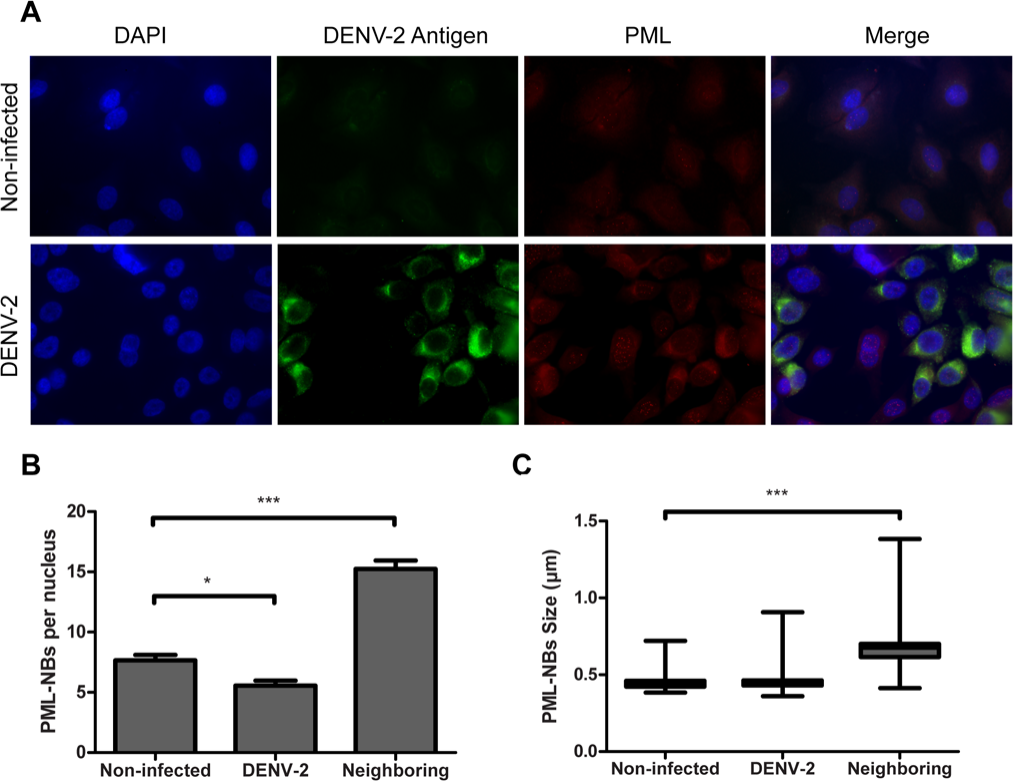
Effect of DENV-2 infection on PML-NBs distribution, number and size. DENV-2 infected A549 cells were fixed 24 h p.i. and a double immunofluorescence staining was performed. (A) Viral antigen expression was visualized by using monoclonal anti-E antibody and FITC-labelled anti-mouse IgG2a. PML was visualized by using anti-PML polyclonal antibodies and TRITC-labelled anti-rabbit IgG1. Cell nuclei were stained with 4',6-diamidino-2-phenylindole (DAPI). Cells were visualized by confocal microscopy. Magnification: 600 X. (B) Quantification of the mean number of PML-NBs in the nuclei of non-infected, DENV-2 infected and DENV-2 infected neighboring A549 cells at 24 h p.i. 50 cell nuclei were counted for each condition. The reported values are mean ± SD. (C) Determination of the mean diameter of PML-NBs in non-infected, DENV-2 infected and DENV-2 infected neighboring A549 cells represented as a box and whiskers plot. The box extends from the 25th percentile to the 75th percentile. The horizontal line represents the median and the whiskers extend down to the smallest value and up to the largest. PML-NB sizes from 100 non-infected, DENV-2 infected or DENV-2 infected neighboring cells were measured. Asterisks indicate a significant difference (*** p < 0.001; ** p < 0.01; * p < 0.05).

More than 90 different proteins can associate with PML-NBs, either transiently or constitutively, but PML is the major structural component of PML-NBs, and their stability depends on PML presence. Several reports have revealed various strategies developed by viruses to interact with PML and, in consequence, to disrupt PML-NBs [3]. Thus, we next examined whether the integrity of PML-NBs was altered and the expression of PML was modified during DENV-2 infection. In order to achieve that, A549 cells were infected with DENV-2 and at 24 h p.i cells were fixed with methanol to detect E viral protein and observe intracellular PML-NBs localization. Double immunofluorescence studies showed a clear decrement in the number of PML-NBs in most DENV-2 infected cells. As can be seen in Fig 3A, in some viral antigen positive cells, the typical punctuate nuclear staining pattern of PML-NBs was lost. This observation implies some degradation of PML-NBs during DENV-2 infection. Moreover, confocal microscopy images showed that neighboring cells presented a weak viral protein signal along with a significantly distinct nuclear staining of PML-NBs, with increased number (15 ± 5 PML-NBs/nucleus) and size (0.7 ± 0.2 μm diameter) with respect to non-infected cells (7 ± 2 PML-NBs/nucleus; 0.5 ± 0.1 μm diameter) (Fig 3B and 3C).

### Dependence of PML expression and distribution with the time of infection

**Fig 4:**
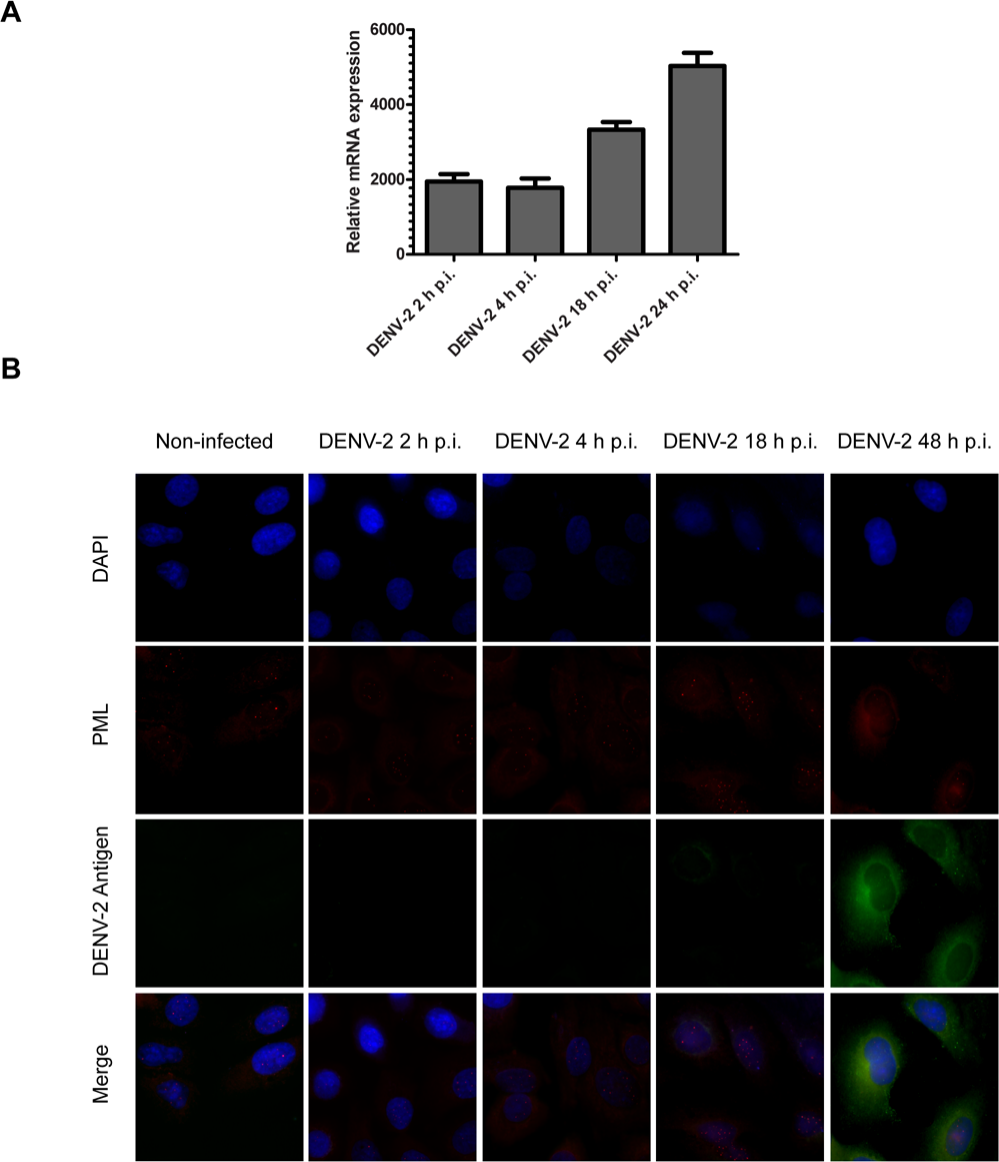
Determination of PML expression with the time of infection. (A) A549 cells were infected with DENV-2 (MOI = 1) and at 2, 4, 16 and 24 h p.i. the expression of PML-mRNA was determined by qRT-PCR. (B) A549 cells were infected with DENV-2 (MOI = 1) and at 2, 4, 18 and 48 h p.i. the expression of PML was revealed by immunofluorescence with anti-PML monoclonal antibodies. Magnification: 400X.

Restriction factors exert their function at different stages of virus life cycle, and their expression varies significantly in different cells and activation states [30]. In order to study the kinetics of PML expression along DENV-2 infection, the PML-mRNA abundance was quantified at 2, 4, 18 and 24 h p.i. by real time PCR. There was a strong correlation between the infection progression and the upregulation of PML-mRNA level. The PML-mRNA abundance in DENV-2 infected A549 cell cultures was significantly higher even at 2 h p.i. and reached a 5000 ± 600 fold increment at 24 h p.i. in comparison to non-infected cells (Fig 4A).

Besides time of infection, the increase in the PML-mRNA levels was dependent on the initial virus stimuli since a higher multiplicity of infection triggered a stronger up-regulation of PML-mRNA at 24 h p.i. (data not shown).

Furthermore, a correlation of protein abundances with the advance of infection was determined by a time course immunofluorescence study. At protein level, it was observed that DENV-2 infected A549 cells showed the typical pattern of PML-NBs at early stages of infection (1–6 h p.i.). However, at late stages (18 h p.i.), when newly formed virions are released from the host cell, some DENV-2 infected A549 infected cells showed a diminished PML-NBs staining but neighboring cells showed a strong anti-PML nuclear signal by monoclonal antibody staining (Fig 4B).

**Fig 5:**
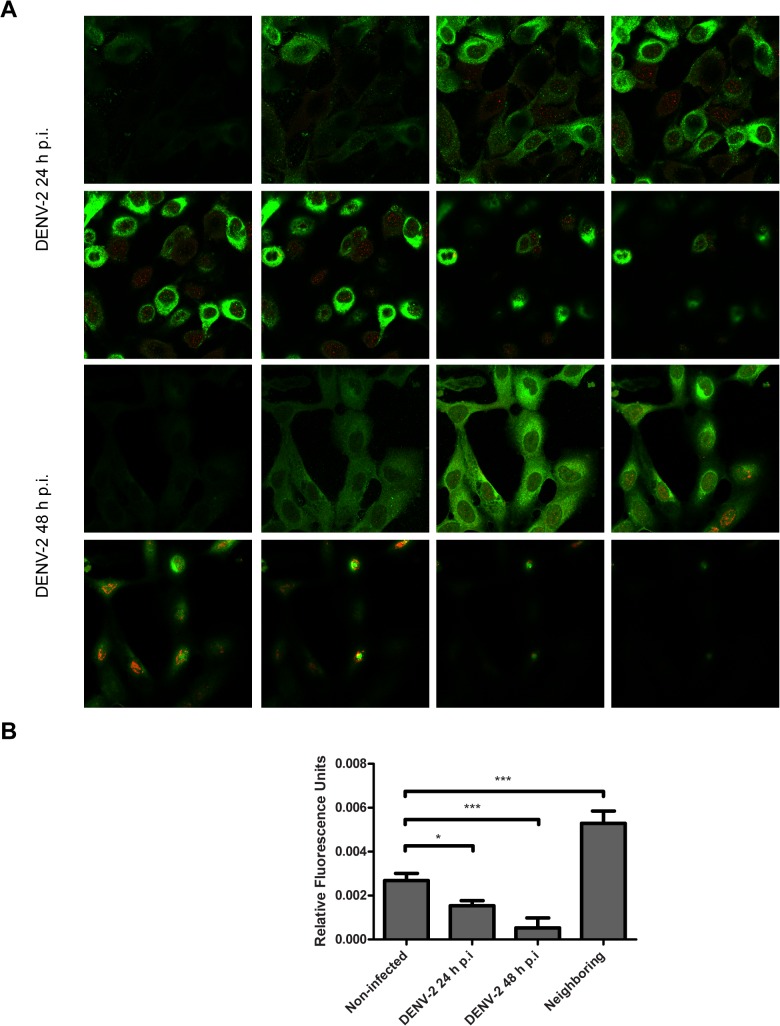
Determination of PML localization during infection. (A) A549 cells were infected with DENV-2 (MOI = 1) and at 24 and 48 h p.i. the expression of PML was revealed by confocal microscopy with anti-PML polyclonal antibodies. Both results are shown in consecutive confocal partial z-stack images. (B) Quantification of cellular PML expression level shown in C.

This observation was clearly confirmed and quantified by confocal studies at 24 h p.i. (Fig 5A and 5B). It is worth mentioning that the pattern of PML-NBs was lost in most of the cells at 48 h p.i., when 100% of the cells were infected (Fig 5A).

### PML anti-DENV-2 activity is enhanced after IFN pathway activation

**Fig 6:**
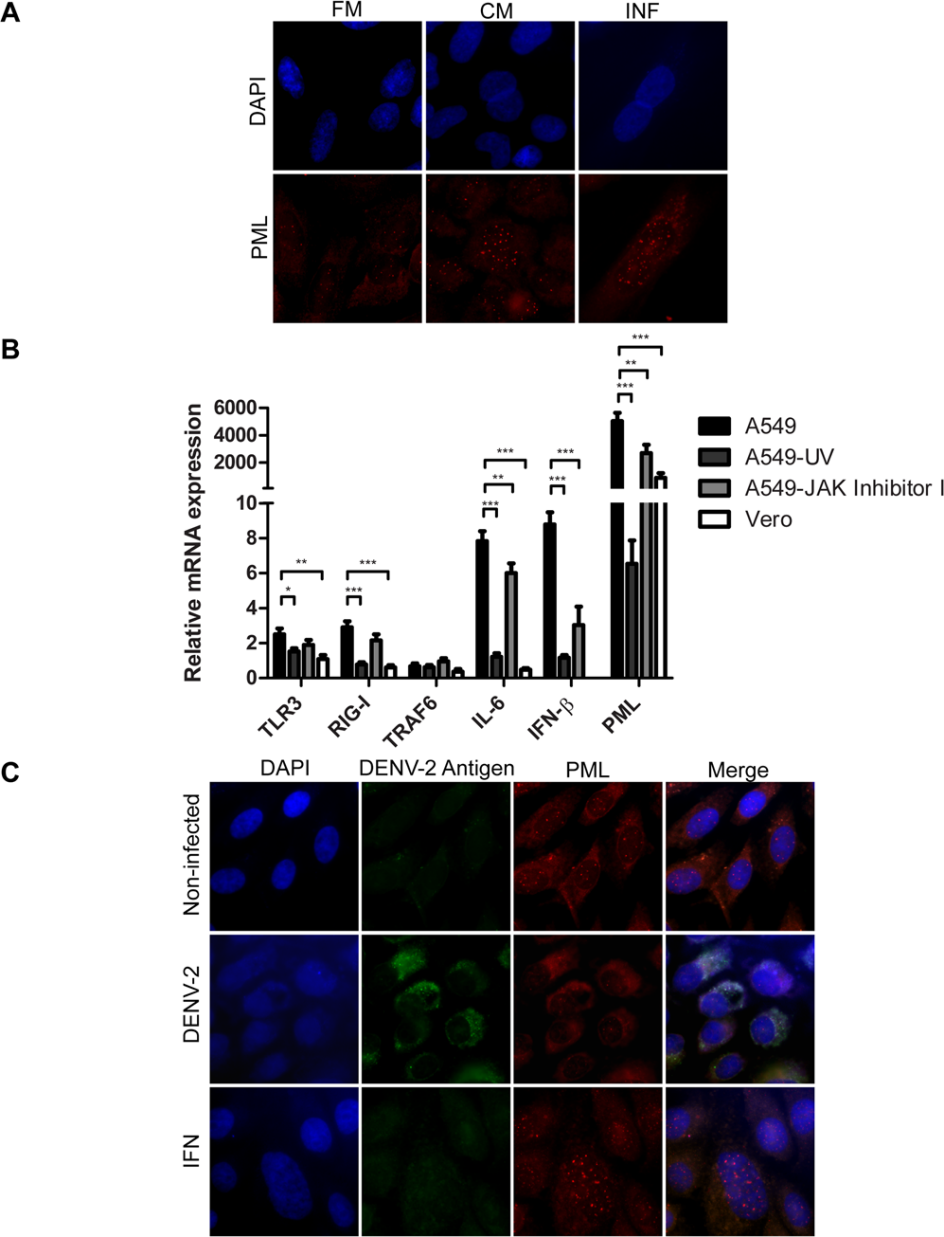
Effect of IFN-I on PML anti-DENV-2 activity. (A) A549 cells were treated with fresh medium (FM), conditioned medium (CM) and medium containing 2500 U/ml of IFN-I during 24 h. Then, cells were fixed and immunostained with anti-PML monoclonal antibodies. (B) A549 and Vero cells were infected with DENV-2 (MOI = 1) and 24 h p.i. the expression of TLR3, RIG-I, TRAF6, IL-6, IFN-b and PML mRNA was determined by real time PCR. A549 cells infected with UV-inactivated DENV-2 (A549-UV) and DENV-2 infected A549 cells treated with 10 μM JAK inhibitor I (A549-JAK inhibitor I) were included for comparison. The mRNA expression level is represented as fold difference relative to mock infected cells and normalized to β-actin-mRNA. (C) Non-infected Vero cells, DENV-2 infected Vero cells and non-infected Vero cells treated with 2500 U/ml of IFN-I (IFN) were incubated during 24 h. Then, cells were fixed and immunostained with anti-PML polyclonal antibodies. Magnification: 600 X.

It is known that IFN-I enhances the intrinsic antiviral activity of PML. Thus, the increment in number and size of PML-NBs in neighboring infected cells, seen in Figs 3 and 4, could have been a response to the IFN-I secreted by the DENV-2 A549 infected cells. In order to assess this possibility, conditioned medium was collected from A549 DENV-2 infected cells and used to treat fresh A549 cells. As positive control, other A549 cell cultures were treated with IFN-I. As it can be seen in Fig 6A, both treatments led to a bigger size and number of PML-NBs in these cell cultures. To gain more insight about this PML-innate immunity related antiviral response, the level of mRNAs of IFN pathway associated genes (TLR3, RIG-I, TRAF6, IFN-β, IL-6 and PML) was quantified in the DENV-2 infected A549 cell cultures at 24 h p.i. As control, UV-inactivated DENV-2 was used for infection, and in parallel, DENV-2 infected A549 cells were treated with an inhibitor of the Janus kinase/signal transducers and activators of transcription (JAK/STAT) pathway to restrict IFN-I signaling. Also Vero cells, a monkey kidney epithelial cell line deficient for IFN production, were infected with DENV-2. In all cases, the same panel of mRNAs IFN pathway related genes was analyzed.

As it is shown in Fig 6B, in A549 DENV-2 infected cells the level of mRNAs corresponding to the dsRNA sensor receptor molecules TLR3 and RIG-I was upregulated, reaching a 2.5 ± 0.3 fold or a 2.9 ± 0.3 fold increase, respectively, in relation to non-infected cells at 24 h p.i. Next, we also included the analysis of expression of the adapter molecule TRAF-6 and two important cytokines IL-6 and IFN-β. While TRAF-6 expression was not altered at this time point of infection, the level of mRNAs corresponding to IL-6 and IFN-β were significantly upregulated in DENV-2 infected A549 cell cultures. It is worth to note that these effects were not observed in non-replicating UV-inactivated DENV-2 infected A549 cell cultures. This response is expected since the IFN potent antiviral response is initiated immediately after the pathogens enter into the cell and begin to replicate. Moreover, the upregulation of IFN pathway related genes was not observed neither in DENV-2 infected A549 cells treated with a JAK-I inhibitor nor in DENV-2 infected Vero cells, confirming that the enhancement was a specific response to the activation of the IFN pathway.

On the other hand, it was observed that PML-mRNA expression level was significantly upregulated in all cell cultures, in comparison with non-infected ones. This observation is in accordance with the constitutive overexpression of PML after virus entry. However, DENV-2 infected A549 cells presented a significant enhancement of PML-mRNA relative expression at 24 h p.i., reaching an increase as high as 5000 ± 600 fold that can be explained by the INF-I stimulation in these cell cultures (Fig 6B).

Lastly, immunoflourescence images showed that in DENV-2 infected Vero cells PML-NBs were also disrupted, but neighboring cells did not present an enhanced PML-NBs pattern. However these cultures showed bigger size and number of PML-NBs after IFN-I treatment (Fig 6C). Altogether these results confirmed the importance of IFN-I to reinforce the intrinsic antiviral activity of PML against DENV-2.

## Discussion and Conclusions

PML-NBs were originally observed through electron microscopy in the early 1960s which revealed the presence of spherical nuclear bodies in most cell types and many tissues [31]. In the last 20 years, extensive studies implicated PML-NBs in stress response [32], gene regulation [33], oncogenesis [34], cell senescence [35], DNA damage repair [36], apoptosis [37] as well as in antiviral defense mechanisms [38].

Intrinsic antiviral resistance is a cellular antiviral defense that involves diverse proteins and mechanisms, depending on the particular virus. Most of the characterized restriction factors have been studied in the context of HIV-1 life cycle and have been described as possessing potent antiretroviral activity *in vitro* and *in vivo* [39–40]. In the last years, it was described that PML is implicated in the intrinsic antiviral immunity against different viruses in the absence of IFN-I, however its activity is enhanced by this molecule [41]. In the present study, we have explored the possible role of PML as an intrinsic restriction factor against DENV-2.

First, we evaluated the impact of PML silencing and overexpression on DENV-2 replication. The silencing of all PML isoforms caused about 0.76 log increment in DENV-2 titre, in comparison with virus production obtained when small non-interfering RNAs were used for transfection. On the other hand, the PMLIV isoform overexpression reduced significantly the extracellular DENV-2 production. These results were in accordance with the viral antigen expression observed by immunofluorescence. The PML silencing rendered A549 cell cultures more susceptible to virus propagation and the overexpression of PMLIV highly inhibited the viral antigen expression in infected cells. It is worth to mention that these results were not influenced by the level of apoptosis in these cell cultures.

Moreover, we analyzed the intracellular localization of PML-NBs during DENV-2 replication. Confocal microscopy images showed that the typical punctuate nuclear staining pattern of PML-NBs was lost during DENV-2 infection. It remains unclear if the disruption of PML-NBs during DENV-2 infection was a consequence of the interaction between a viral component and this restriction factor, as a viral counterpart action against this antiviral cellular activity. Furthermore, confocal images showed a weak viral protein signal in neighboring cells, which also displayed increased number and size of PML-NBs.

The next step was to determine the abundance of PML-mRNA at different stages of DENV life cycle. We confirmed that there is a strong correlation between the infection progression and the upregulation of PML-mRNA level. However, when time course immunofluorescence studies were performed, the pattern of PML did not change during the early stages of infection, and only after the new progeny of DENV-2 was released, a reduction in PML-NBs staining was observed. Altogether these results strongly suggest that a viral protein might be responsible of the PML-NBs disruption. This phenomenon could be a consequence of these protein interactions, where PML could be sequestrated by a DENV protein. Several proteins have been reported to interact with nuclear components, including factors related to IFN response [42–44]. Another speculation might be a competition for SUMOylation between a viral protein and PML, as it was described that PML-NBs assembly strictly depends on this post-translational modification [45].

Early studies indicated that many PML-NBs proteins, such as PML and Sp100, are IFN inducible [46]. IFN-I treatment of cells greatly increases the number, intensity and size of PML-NBs and thus, this contributes to an enhanced antiviral innate response of PML [8, 9]. In order to confirm that the increment in number and size of PML-NBs in neighboring cells was related to the IFN-I secretion, medium collected from infected cells was used to treat fresh A549 cell cultures. In these treated uninfected cells, a huge increment in size and number of PML-NBs was seen; confirming the presence of IFN-related soluble factors in the collected medium from DENV-2 infected A549 cells. Furthermore, the mRNA level of the central molecules of innate immune pathway was analyzed. The mRNAs corresponding to IL-6 and IFN-β were significantly up-regulated in DENV-2 infected A549 cell cultures, and this effect was not observed neither in non-replicating UV-inactivated DENV-2 infected A549 cell cultures nor in DENV-2 infected cells in the absence of IFN. Interestingly, the PML-mRNA expression level was significantly upregulated in all cell cultures, in comparison with non-infected cells. However, A549 DENV-2 infected cells presented a significant enhancement of PML-mRNA relative expression reaching an increase as high as 5000-fold.

These results confirmed that PML intrinsic antiviral mechanism is independent of IFN, however, it is noticeably enhanced in IFN producing cell lines (A549) in comparison to cell lines deficient for IFN production (Vero), showing the importance of PML also in the innate immune response. Thus, it seems that the constitutive expression of PML would allow for immediate antiviral activity of this molecule, an effect that is potentiated in neighboring cells upon upregulation through IFN produced by the infected cells, or it would indirectly participate in the IFN-mediated upregulation of known or yet unknown IFN-regulated proteins with antiviral activity.

In conclusion, several genome-wide RNAi screens have identified host enhancement or host restriction factors involved in the viral replication cycle of several members of the *Flaviviridae* family including hepatitis C virus (HCV) [47–48], West Nile virus (WNV) [49], and Yellow fever virus (YFV) [50]. However, limited studies about human host restriction factors involved in DENV replication have been described [51].

The present study constitutes the first evidence that PML contributes to a cellular antiviral response and restricts DENV-2 replication in human A549 cells. The exact mechanism behind PML-mediated intracellular defense against DENV, as well as the strategy of virus evasion from this cellular defense requires further study. Furthermore, the evaluation of the antiviral properties of PML in other susceptible cell types more representative of the natural infection will improve the perspectives for a future therapeutic use as anti-DENV target. Moreover, the importance of PML antiviral activity for other viruses has been shown also in animal models. PML deficiency renders mice more susceptible to some viral infections [52], resulting in an increased viral replication. Despite that our *in vitro* results are in accordance with the findings of these groups, it would be necessary to demonstrate in future studies the relevance of PMLIV anti-DENV activity *in vivo*. It would be interesting to investigate if the kinetics of this host restriction factor at different stages of human DENV infection could be used as a biomarker in disease progression or in the assessment of the severity of dengue disease.
