# Correction: Cellular Promyelocytic Leukemia Protein Is an Important Dengue Virus Restriction Factor

**DOI:** 10.1371/journal.pone.0137040

**Published:** 2015-08-27

**Authors:** Federico Giovannoni, Elsa B. Damonte, Cybele C. García

In Table 1, the PML primer sequence is recorded incorrectly.

Please see the corrected table here:

**Table 1:** Primer sequences used for real-time RT-PCR assays.

Primer	Orientation	Sequence
TLR3	Sense	5′-AGTGCCCCCTTTGAACTCTT-3′
	Antisense	5′-ATGTTCCCAGACCCAATCCT-3′
RIG-I	Sense	5′-TGTTCTCAGATCCCTTGGATG-3′
	Antisense	5′-CACTGCTCACCAGATTGCAT-3′
TRAF6	Sense	5′-TGCCATGAAAAGATGCAGAG-3′
	Antisense	5′-AAGGCGACCCTCTAACTGGT-3′
IL-6	Sense	5′-TGTGAAAGCAGCAAAGAGGCACTG-3′
	Antisense	5′-ACAGCTCTGGCTTGTTCCTCACTA-3′
IFN-β	Sense	5′-TAGCACTGGCTGGAATGAGA-3′
	Antisense	5′-TCCTTGGCCTTCAGGTAATG-3′
PML	Sense	5´-CTTGCATCACCCAGGGGAAA-3´
	Antisense	5’-CCGGTTCACCGCAGCCAGCT-3´

## References

[1] Giovannoni F, Damonte EB, García CC (2015) Cellular Promyelocytic Leukemia Protein Is an Important Dengue Virus Restriction Factor. PLoS ONE 10(5): e0125690 doi:10.1371/journal.pone.0125690 25962098 10.1371/journal.pone.0125690 PMC4427460

